# Impact of Well-Controlled Type 2 Diabetes on Corneal Endothelium Following Cataract Surgery: A Prospective Longitudinal Analysis

**DOI:** 10.3390/jcm14103603

**Published:** 2025-05-21

**Authors:** Aleksandra Opala, Łukasz Kołodziejski, Iwona Grabska-Liberek

**Affiliations:** Department of Ophthalmology, Centre of Postgraduate Medical Education, 01-813 Warsaw, Poland

**Keywords:** phacoemulsification, cataract surgery, corneal endothelium, endothelial cell density (ECD), type 2 diabetes, well-controlled diabetes, hexagonal cell percentage (%HEX), cell size variability (CV), central corneal thickness (CCT), cumulative dissipated energy (CDE)

## Abstract

**Background:** The aim of this study was to evaluate corneal endothelial changes following phacoemulsification cataract surgery with intraocular lens implantation in patients with type 2 diabetes (study group) and without diabetes (control group). The study aimed to determine the extent of endothelial cell damage and the regenerative capacity of the cornea in patients with well-controlled diabetes. **Methods:** This study compared corneal endothelial parameters in 80 eyes (80 patients) with well-controlled type 2 diabetes and 80 eyes (80 patients) without diabetes, all of whom underwent uneventful phacoemulsification cataract surgery. Patients were examined preoperatively and at 14 days, 3 months, and 6–8 months postoperatively. Endothelial cell density (ECD), percentage of hexagonal cells (%HEX), cell size variability (CV), and central corneal thickness (CCT) were assessed using a specular microscope. Visual acuity, intraocular pressure (IOP), and cumulative dissipated energy (CDE) during phacoemulsification were also measured. **Results:** The study and control groups were matched for age and sex. Preoperatively, patients with type 2 diabetes had significantly lower endothelial cell density (2480.76 ± 303.48 cells/mm^2^) compared to the control group (2629.64 ± 304.73 cells/mm^2^, *p* = 0.002). Visual acuity was also significantly lower in the study group (0.44 ± 0.18) than in the control group (0.50 ± 0.19, *p* = 0.049). No significant preoperative differences were observed in IOP, CV, %HEX, or CCT. Postoperatively, both groups experienced ECD decline: −18.44%, −18.77%, and −19.05% in the study group and −15.12%, −16.42%, and −16.73% in the control group at 14 days, 3 months, and 6–8 months, respectively. Differences between groups were not statistically significant (*p* = 0.285). A significant %HEX decrease was observed in both groups at all time points, with a greater decline in the study group at 14 days and 3 months. CV significantly increased in both groups at 14 days and 3 months postoperatively, but no significant difference was found between groups. A significant increase in CCT was observed at 14 days and 3 months postoperatively, with a greater increase in the study group at 14 days. Preoperative visual acuity negatively correlated with CDE in both groups. Additionally, CDE negatively correlated with ECD at all time points. **Conclusions:** Endothelial cell density is lower in patients with well-controlled type 2 diabetes than in non-diabetic individuals. Both groups are at risk of endothelial cell loss during phacoemulsification. Despite good glycemic control and comparable preoperative endothelial morphology, the cornea in diabetic patients is more vulnerable to damage, with a prolonged regeneration process. The impaired regenerative capacity of the corneal endothelium suggests the need for additional precautions during cataract surgery in diabetic patients. Despite ECD decline and delayed endothelial regeneration, the functional status of the cornea, as indicated by visual acuity and CCT, remains stable. The adequate corneal endothelial cell reserve in well-controlled type 2 diabetes patients allows for cataract surgery without significant corneal complications.

## 1. Introduction

Cataract is one of the leading causes of visual impairment in patients with diabetes [1,2,3]. There are reports that cataract surgery using the phacoemulsification method leads to a decrease in corneal endothelial cell density [4,5,6,7]. Permanent impairment of corneal endothelial function may result in persistent corneal edema, corneal decompensation, and the development of bullous keratopathy [8].

According to the literature, the baseline number of corneal endothelial cells in the group of diabetic patients is reduced [9,10,11]. Some researchers suggest that endothelial cell loss during cataract surgery in diabetic patients is greater than in non-diabetic patients [5,12].

On the other hand, diabetic complications increase the risk of complications during cataract surgery. These patients more frequently exhibit a weakened pupillary response to mydriasis, which results from ischemia and impaired parasympathetic innervation of the pupil [13]. In this patient group, mechanical methods of pupil dilation are more commonly used during cataract surgery, and the duration of the procedure is often prolonged.

Due to the multifaceted relationships between both conditions, the decision to undertake surgical treatment in this patient group must be made with caution. It is widely recognized that cataract phacoemulsification in diabetic patients is a high-risk procedure.

Changes in the morphology and number of corneal endothelial cells, described by parameters such as endothelial cell density (ECD), coefficient of variation in cell area (CV), and hexagonality index (%HEX), affect the ability of the endothelium to function properly as a protective barrier for the corneal stroma. Abnormal endothelial cell morphology, combined with increased central corneal thickness (CCT), serves as a marker of endothelial dysfunction, leading to fluid imbalance, stromal edema, and loss of corneal transparency, thereby impairing vision.

Factors related to the course of cataract phacoemulsification have a detrimental impact on endothelial cell status. These include the hardness of the lens nucleus, which determines the energy and effective duration of phacoemulsification, as well as mechanical maneuvers associated with lens fragmentation and intraocular lens implantation. These factors, combined with the compromised condition of endothelial cells in diabetes, indicate an increased risk of long-term endothelial cell dysfunction, potentially leading to decompensation and the development of bullous keratopathy [5,12].

This study aimed to investigate potential differences in corneal endothelial structure and central corneal thickness (CCT) between well-controlled type 2 diabetic patients and non-diabetic individuals who underwent uneventful cataract surgery.

## 2. Materials and Methods

### 2.1. Patient Characteristics

The study participants were recruited from patients presenting at the Department of Ophthalmology and the associated outpatient clinic. The patients were divided into two groups:

1. Study group—patients who underwent cataract surgery using the phacoemulsification method and had a diagnosis of well-controlled type 2 diabetes (HbA1c ≤ 7%).
2. Control group—patients who underwent cataract surgery using the phacoemulsification method and did not have type 2 diabetes.

Each group consisted of 80 patients, with a total of 160 patients participating in the study. Data from one eye of each patient were analyzed, resulting in 160 eyes included in the study. The study included patients aged 60 to 80 years. The diagnosis of type 2 diabetes was based on a medical history assessment and written confirmation from the treating physician. All diabetic patients were receiving oral antidiabetic medications during the study period. The duration of type 2 diabetes ranged from 1 to 10 years. All patients were of Caucasian ethnicity. For all patients with type 2 diabetes, glycated hemoglobin (HbA1c) levels were measured in serum to assess glycemic control. To extrapolate the average glycemic control over at least 6 months, two HbA1c samples were collected at a 3-month interval: the first sample was taken before study inclusion, and the second sample was taken 3 months after surgery. In the diabetic patient group, either no diabetic retinopathy was present or only mild nonproliferative diabetic retinopathy was observed. No patient included in the study had moderate or severe nonproliferative diabetic retinopathy or proliferative diabetic retinopathy. The assessment and classification of diabetic retinopathy stages were conducted using the Early Treatment of Diabetic Retinopathy Study (ETDRS) scale. None of the patients had a history of prior retinal photocoagulation or treatment for diabetic macular edema with anti-VEGF agents. Non-diabetic patients who underwent surgery during the same period were included in the study as the control group. Fasting blood glucose levels were measured in accordance with American Diabetes Association (ADA) guidelines to detect any previously undiagnosed diabetes. All patients included in the study had age-related cataracts at a stage qualifying for surgical treatment, assessed using the Lens Opacities Classification System III (LOCS III) (Table 1).

### 2.2. Research Methods

At the time of inclusion in the study, the patient’s age and gender were recorded, and a medical history interview was conducted, including information on the duration of type 2 diabetes, type of treatment, and the presence of comorbid conditions.

All patients underwent a biomicroscopic examination to assess the anterior segment of the eye. The severity of cataracts was evaluated using the LOCS III scale. The posterior segment and the fundus of the eye were examined based on the Diabetic Retinopathy Classification. Best corrected visual acuity (BCVA) was measured using Snellen charts, and intraocular pressure (IOP) was assessed using Goldmann applanation tonometry. Each patient underwent ocular biometry using ultrasound biometry (Alcon OcuScan RxP Measuring System), recording measurements of axial length (AXL), anterior chamber depth (ACD), and lens anterior-posterior dimension (L).

Corneal endothelial cell density (ECD) (cells/mm^2^) in the central area, coefficient of variation in endothelial cell size (CV), percentage of hexagonal cells (%HEX), and central corneal thickness (CCT) were analyzed using a non-contact specular microscope (Nidek CEM-530, Nidek Co., Ltd., Gamagori, Japan). During each visit, three images of each cornea were taken. For further analysis, one image of satisfactory quality and an average ECD value compared to the other two measurements was selected.

Cataract removal surgeries were performed by four experienced surgeons using the Centurion Vision System (Alcon Laboratories, Inc., Fort Worth, TX, USA). A standard phacoemulsification cataract removal protocol was applied (using the stop and chop technique) with implantation of an intraocular lens (a single-piece foldable AcrySof lens, model SN60AT, Alcon). Each procedure was uneventful. Intraoperatively, the cumulative dissipated energy (CDE) was recorded. According to the manufacturer, cumulative dissipated energy (CDE) is defined as the total ultrasound energy in pedal position 3 (longitudinal and torsional) calculated as follows: (longitudinal power time × average longitudinal power) + (torsional power time × 0.4 × average torsional amplitude). The coefficient of 0.4 corresponds to an approximate reduction in dissipated heat during incision compared to conventional phacoemulsification.

The postoperative topical treatment protocol included levofloxacin at a dose of one drop into the conjunctival sac four times daily for 7 days, dexamethasone 0.1% in a gradually decreasing dose over 4 weeks (starting with four times daily, reducing by one dose per day every 7 days), tropicamide 1% once daily for 7 days, nepafenac three times daily for 4 weeks, and a preservative-free moisturizing preparation four times daily.

Postoperative follow-up examinations were conducted at the following time points: 2 weeks, 3 months, and between 6 and 8 months after surgery. All examinations were performed by a single investigator. At each follow-up visit, all patients underwent a biomicroscopic examination using a slit lamp to assess the anterior segment of the eye. Corneal endothelial parameters were measured using a non-contact specular microscope (Nidek CEM-530, Nidek Co., Ltd., Gamagori, Japan), evaluating ECD, CV, and %HEX. CCT was measured simultaneously using the specular microscope. Best corrected visual acuity and intraocular pressure (IOP) were assessed using Goldmann applanation tonometry (Haag-Streit AT 900 Goldmann Applanation Tonometer, Köniz, Switzerland).

The study protocol was approved by the Bioethics Committee on 15 June 2020 (resolution number 97/PB/2020). Informed consent was obtained from each patient participating in the study.

### 2.3. Statistical Analysis

Statistical analysis was conducted using R software (version 4.1.2). To describe gender distribution, absolute and relative frequencies were reported. Quantitative parameters were described using the mean and standard deviation, median and interquartile range (first and third quartiles), as well as the range (minimum and maximum values). The conformity of numerical parameter distributions with the normal distribution was verified using the Shapiro-Wilk test. If the Shapiro-Wilk test indicated a lack of normality, additional verification was performed using skewness and kurtosis coefficients. In such cases, the distribution was considered normal if the skewness coefficient was between −1 and 1 and the kurtosis was between 2 and 4, meaning that both indicators suggested a shape similar to the normal distribution. Homogeneity of variance was assessed using Levene’s test.

The groups of patients with and without diabetes were compared using the following statistical tools:

- Student’s *t*-test for independent groups, applied when the parameter distribution in both groups conformed to a normal distribution; in all such cases, homogeneity of variance was confirmed,
- Mann–Whitney U test, applied when the assumption of normal distribution was not met for both groups,
- Pearson’s chi-square test, used for gender comparison between groups.

The statistical significance of changes in parameter values over time (relative to baseline measurement) was assessed using the following statistical tools:

- Paired *t*-test, applied when the difference between measurements followed a normal distribution,
- Wilcoxon test, applied when the assumption of normality of differences between measurements was not met.

Differences in quantitative parameters between groups or between measurements were described using the difference in means or medians (depending on normality of distributions), along with a 95% confidence interval. Relationships between two quantitative parameters were analyzed using Spearman’s correlation, given that some parameters exhibited a distribution that deviated from normality.

In all statistical analyses, the significance level was set at alpha = 0.05.

## 3. Results

### 3.1. Characteristics of the Analyzed Patient Groups

The study group consisted of N = 80 patients diagnosed with type 2 diabetes who underwent cataract surgery using the phacoemulsification method (DM+). The control group consisted of N = 80 non-diabetic patients who also underwent cataract surgery using the phacoemulsification method (DM−). The characteristics of the analyzed groups are presented in Table 2 and Table 3, respectively.

### 3.2. Comparison of Groups

The groups were compared in terms of characteristics, biometric parameters, and corneal endothelium parameters in the baseline measurement. It was determined that the groups did not differ significantly in terms of gender and age (*p* > 0.999 and *p* = 0.155, respectively). No statistically significant differences were found between the biometric parameters (*p* > 0.05 in each case).

It was established that the groups differed significantly in the parameter characterizing visual acuity (vis) in the baseline measurement, with the value of this parameter being 0.06 lower in the diabetic patient group, MD = −0.06, CI95 [−0.11; 0.00], *p* = 0.049. It is worth noting that the *p*-value, resulting from the statistical test verifying the significance of the difference in visual acuity between the groups, was at the threshold of statistical significance (the statistical significance threshold is alpha = 0.05).

Furthermore, a significant difference was noted in endothelial cell density (ECD) in the baseline measurement, which was lower in the study group by 148.88 cells/mm^2^, MD = −148.88, CI95 [−243.84; −53.91], *p* = 0.002. Other corneal endothelial parameters, intraocular pressure, central corneal thickness, and mean dissipated energy did not differentiate the groups in the baseline measurement (*p* > 0.05 in each case), as shown in Table 4.

### 3.3. Characteristics of Selected Ophthalmic Parameters in the Study and Control Groups at Different Time Points

For each of the analyzed parameters (Vis, ECD, CV, %HEX, CCT), measurements were taken before surgery, as well as 14 days, 3 months, and 6–8 months after surgery. To understand changes in these parameters over time, comparisons were made between each postoperative measurement and the baseline measurement. The results for the group of patients with type 2 diabetes are presented in Table 5, while the results for patients without type 2 diabetes are shown in Table 6.

#### 3.3.1. Visual Acuity (Vis)

**Figure 1:**
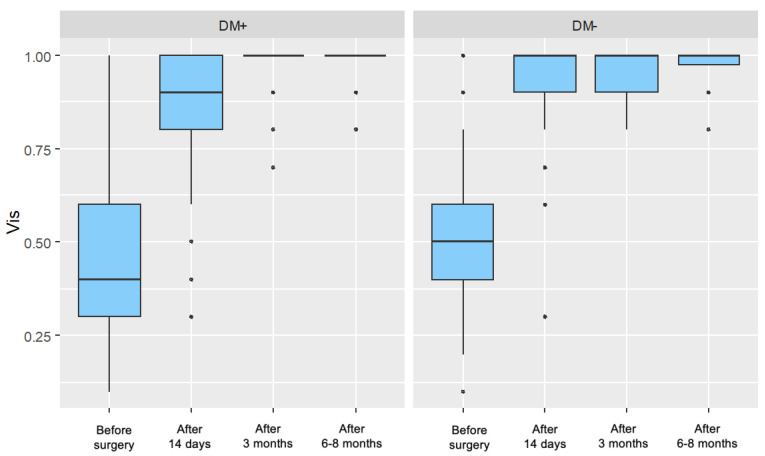
Distribution of Visual Acuity (Vis) for Each Measurement in the Study Group (DM+) and Control Group (DM−).

For both groups, a significant increase in visual acuity was observed in each postoperative measurement compared to the preoperative measurement, with these improvements becoming more pronounced over time. Figure 1 presents the distribution of visual acuity values, divided by individual measurements and the two analyzed patient groups.

#### 3.3.2. Endothelial Cell Density (ECD)

**Figure 2:**
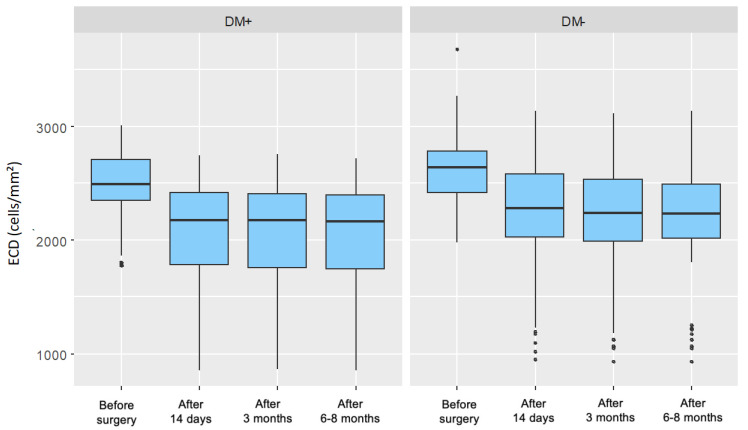
Distribution of Endothelial Cell Density (ECD) for Each Measurement in the Study Group (DM+) and Control Group (DM−).

For both groups, a significant decrease in endothelial cell density was observed in each postoperative measurement compared to the preoperative measurement. In the control group, these decreases became progressively more pronounced over time, whereas in the group of patients with type 2 diabetes, no similar trend was observed. Figure 2 presents the distribution of endothelial cell density, divided by individual measurements and the two analyzed patient groups.

#### 3.3.3. Cell Variability Coefficient (CV)

**Figure 3:**
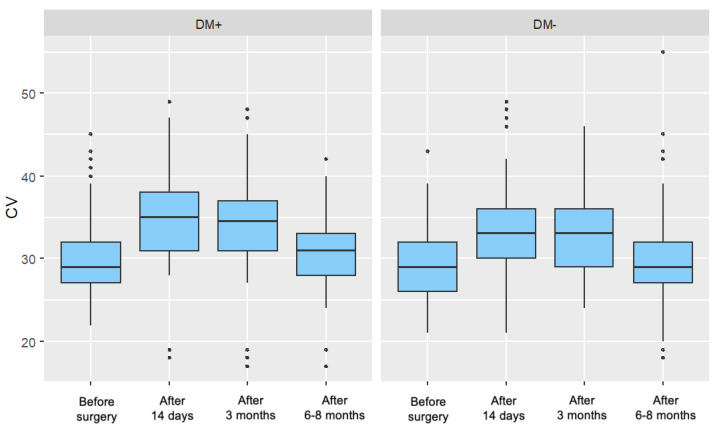
Distribution of the Cell Variability Coefficient (CV) for Each Measurement in the Study Group (DM+) and Control Group (DM−).

For the group of patients with type 2 diabetes, a significant increase in the parameter value was observed in each postoperative measurement compared to the preoperative measurement. These increases gradually diminished over time after surgery. For the control group, a significant increase in the parameter value was recorded 14 days and 3 months after surgery. However, in the measurement taken 6–8 months postoperatively, no significant difference in the cell variability coefficient was observed compared to the preoperative measurement. Figure 3 presents the distribution of the cell variability coefficient, divided by individual measurements and the two analyzed patient groups.

#### 3.3.4. Percentage of Hexagonal Cells (%HEX)

**Figure 4:**
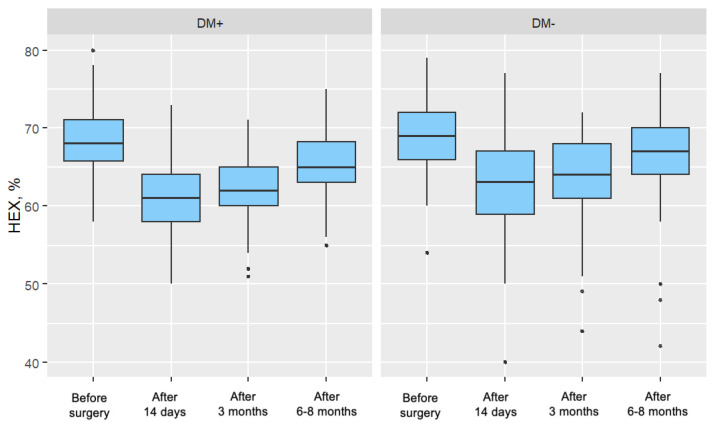
Distribution of the Percentage of Hexagonal Cells (%HEX) for Each Measurement in the Study Group (DM+) and Control Group (DM−).

For both groups, a significant decrease in the parameter value was observed in each postoperative measurement compared to the preoperative measurement. In both groups, these decreases became progressively weaker over time after surgery. Figure 4 presents the distribution of the percentage of hexagonal cells, divided by individual measurements and the two analyzed patient groups.

#### 3.3.5. Central Corneal Thickness (CCT)

**Figure 5:**
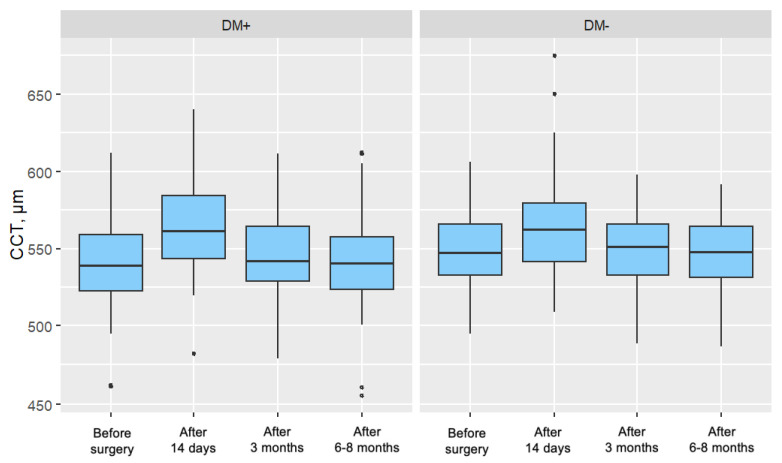
Distribution of Central Corneal Thickness (CCT) for Each Measurement in the Study Group (DM+) and Control Group (DM−).

In both groups, a significant increase in CCT was observed 14 days and 3 months after surgery, with these increases gradually diminishing over time. In the measurement taken 6–8 months postoperatively, no significant difference in central corneal thickness was noted compared to the preoperative measurement for either group. Figure 5 presents the distribution of central corneal thickness, divided by individual measurements and the two analyzed patient groups.

### 3.4. Comparison of Groups in Terms of Parameter Values at Different Time Points

The groups were compared regarding the values of measured parameters across all time points.

#### 3.4.1. Intraocular Pressure (IOP)

It was determined that the groups did not significantly differ in intraocular pressure (IOP) values at any of the measurements (*p* > 0.05) (Table 7).

#### 3.4.2. Visual Acuity (Vis)

A statistically significant difference in visual acuity (Vis) was observed at the baseline measurement, with the value being 0.06 lower in the group of diabetic patients, MD = −0.06, CI95 [−0.11; 0.00], *p* = 0.049. It is noteworthy that the *p*-value from the statistical test verifying the significance of the Vis difference between groups was at the threshold of statistical significance (the statistical significance threshold is alpha = 0.05) (Table 7).

#### 3.4.3. Endothelial Cell Density (ECD)

A significant difference in ECD was noted at the baseline measurement and in all follow-up measurements. ECD was lower in the study group than in the control group at each measurement (before surgery: MD = −148.88, CI95 [−243.84; −53.91], *p* = 0.002; after 14 days: MD = −209.17, CI95 [−371.90; −46.45], *p* = 0.012; after 3 months: MD = −183.85, CI95 [−347.58; −20.12], *p* = 0.028; after 6–8 months: MD = −182.09, CI95 [−345.65; −18.52], *p* = 0.029) (Table 7).

#### 3.4.4. Cell Variability Coefficient (CV)

It was determined that the groups differed significantly in CV at the 14-day measurement, with the value being 2.00 higher in the diabetic patient group, MD = 2.00, CI95 [0.00; 3.00], *p* = 0.035 (Table 7).

#### 3.4.5. Percentage of Hexagonal Cells (%HEX)

A significant difference in %HEX was observed in all postoperative measurements. The %HEX in the study group was lower by 2.00 percentage points compared to the control group for each of these measurements (after 14 days: MD = −2.00, CI95 [−4.00; −46.45], *p* = 0.016; after 3 months: MD = −2.00, CI95 [−3.00; −0.00], *p* = 0.011; after 6–8 months: MD = −2.00, CI95 [−3.00; 0.00], *p* = 0.010) (Table 7).

#### 3.4.6. Central Corneal Thickness (CCT)

It was determined that the groups did not significantly differ in CCT at any of the measurements (*p* > 0.05) (Table 7).

### 3.5. Analysis of Changes in Parameter Values at Different Time Points

Changes in the analyzed parameters between the follow-up measurement and the baseline measurement for patients with type 2 diabetes are presented in Table 8, while for patients without type 2 diabetes, in Table 9. Based on these data, a comparison was made between patients with diabetes (DM+) and without diabetes (DM−) regarding the difference in the magnitude of changes in the analyzed parameters relative to the baseline measurement, which is presented in Table 10.

#### 3.5.1. Visual Acuity (Vis)

A significant difference between the groups was observed in the change in Vis between the preoperative measurement and the measurement taken 3 months after surgery, as well as between the preoperative measurement and the measurement taken 6–8 months after surgery. In the diabetes group, Vis was 0.06 lower than in patients without diabetes, MD = −0.06, CI95 [−0.12; −0.01], *p* = 0.029, and MD = −0.06, CI95 [−0.11; −0.00], *p* = 0.048, respectively. No significant difference between the groups was noted in the change between the preoperative measurement and the measurement taken 14 days after surgery (*p* = 0.489) (Table 10).

#### 3.5.2. Endothelial Cell Density (ECD)

No significant differences between the groups were observed in the change between the preoperative measurement and the follow-up measurement at any visit (*p* > 0.05) (Table 10).

#### 3.5.3. Coefficient of Variation of Cell Size (CV)

No significant differences between the groups were observed in the change between the preoperative measurement and the follow-up measurement at any visit (*p* > 0.05) (Table 10).

#### 3.5.4. Percentage of Hexagonal Cells (%HEX)

A significant difference between the groups was observed in the change in %HEX between the preoperative measurement and the measurement taken 14 days after surgery, as well as between the preoperative measurement and the measurement taken 3 months after surgery. In the diabetes group, %HEX was higher by 1.00 pp and 2.00 pp compared to patients without diabetes, MD = 1.00, CI95 [0.00; 2.00], *p* = 0.021, and MD = 2.00, CI95 [0.00; 2.00], *p* = 0.012, respectively. No significant difference between the groups was noted in the change between the preoperative measurement and the measurement taken 6–8 months after surgery (*p* = 0.106) (Table 10).

#### 3.5.5. Central Corneal Thickness (CCT)

A significant difference between the groups was observed in the change in CCT between the preoperative measurement and the measurement taken 14 days after surgery, where CCT in the diabetes group was 9.00 μm higher than in patients without diabetes, MD = −9.00, CI95 [−11.00; −4.00], *p* < 0.001. No significant difference between the groups was noted in the change between the preoperative measurement and the measurement taken 3 months after surgery, as well as between the preoperative measurement and the measurement taken 6–8 months after surgery (*p* = 0.055 and *p* = 0.177, respectively) (Table 10).

### 3.6. Characteristics of Endothelial Cell Density (ECD) Changes at Different Time Points

**Figure 6:**
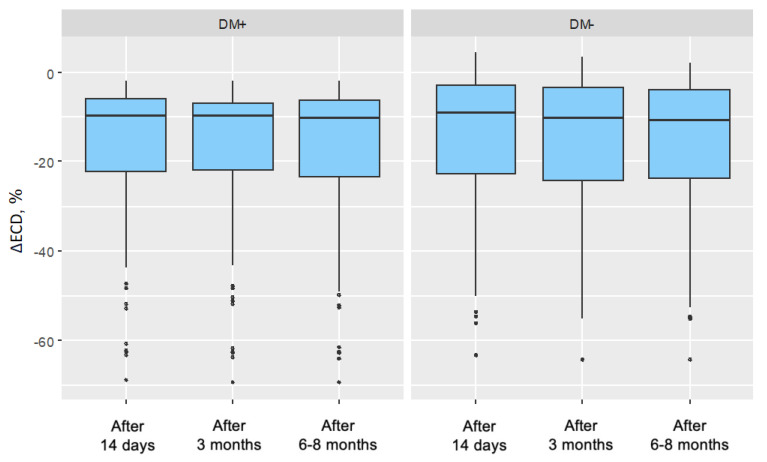
Distributions of endothelial cell density (ECD) change in the group of patients without diabetes for each measurement in the study group (DM+) and control group (DM−) (ECD change in percentage values was referenced to the baseline measurement).

The change in endothelial cell density at specific follow-up points was compared to the baseline measurement and expressed as a percentage change. In all follow-up measurements and in both groups, endothelial cell density decreased relative to the preoperative measurement. The mean changes in endothelial cell density showed a lower negative value in the group of patients with type 2 diabetes compared to the corresponding changes in the control group. This indicated that the decreases in endothelial cell density were more pronounced in the group of patients with type 2 diabetes compared to the changes in the control group (Table 11). The distributions of endothelial cell density changes are illustrated in Figure 6.

### 3.7. Analysis of Correlation Between Individual Parameters in the Study Group and the Control Group

#### 3.7.1. Correlation Analysis Between Cumulative Dispersed Energy (CDE) and Individual Parameters

For each group, an analysis was conducted to determine whether a correlation existed between cumulative dispersed energy (CDE) and the individual parameters included in the analysis. Additionally, the direction and strength of any observed correlation were assessed.

Spearman’s correlation analysis was used for this purpose, as some parameters exhibited distributions deviating from a normal distribution. The results are presented in Table 12.

#### 3.7.2. Visual Acuity (Vis)

**Figure 7:**
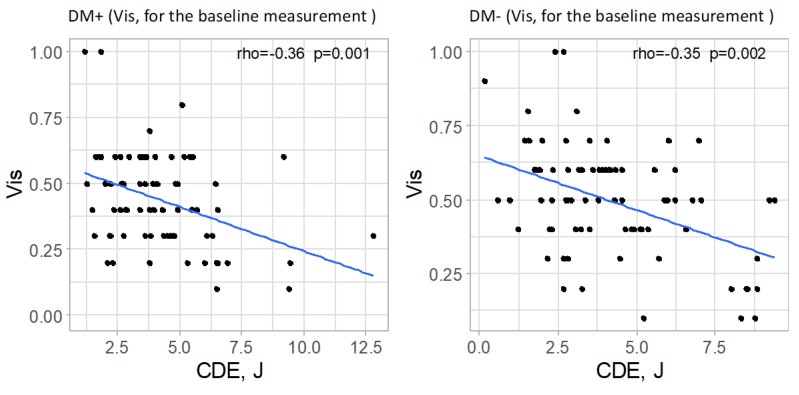
Significant relationships between cumulative dispersed energy (CDE) and visual acuity (Vis, for the baseline measurement) in both groups.

In both analyzed groups, a significant correlation was observed between cumulative dispersed energy (CDE) and visual acuity in the baseline measurement (*p* = 0.001 and *p* = 0.002 for the study and control groups, respectively). The strength of these correlations was moderate, with a negative direction (correlation coefficient rho = −0.36 and rho = −0.35, respectively), indicating that higher ultrasound energy values were associated with lower preoperative visual acuity. In the postoperative measurements, no significant correlations were found between the analyzed parameters in either group (*p* > 0.05). Significant correlations are illustrated in Figure 7.

#### 3.7.3. Endothelial Cell Density (ECD)

In the group of patients with type 2 diabetes, a significant correlation was observed between cumulative dispersed energy (CDE) and endothelial cell density in each postoperative measurement (*p* < 0.001 for each measurement). The strength of these correlations was moderate in all postoperative measurements, and the direction was negative (rho = −0.41, rho = −0.41, and rho = −0.42, respectively, for successive measurements). This indicated that higher values of cumulative dispersed energy were associated with lower endothelial cell density.

**Figure 8:**
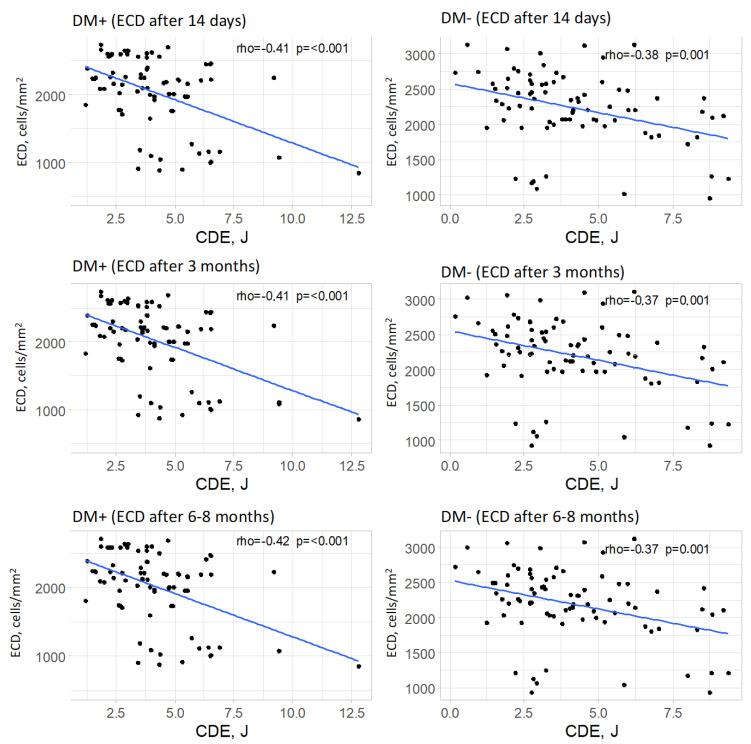
Relationships between cumulative dispersed energy (CDE) and endothelial cell density (ECD, for all measurements) in both groups.

In the control group, a significant correlation was also found between cumulative dispersed energy and endothelial cell density in postoperative measurements (*p* = 0.001 for each postoperative measurement). The strength of these correlations was moderate, and the direction was negative (rho = −0.38, rho = −0.37, and rho = −0.37, respectively, for successive postoperative measurements). This suggested that higher values of cumulative dispersed energy were associated with lower endothelial cell density, with a moderate correlation strength. The relationships between cumulative dispersed energy and endothelial cell density are visually represented for each measurement and for both groups in Figure 8.

#### 3.7.4. Coefficient of Variation of Cells (CV)

No significant correlations were observed for any of the measurements and for any of the analyzed groups (*p* > 0.05 in all cases).

#### 3.7.5. Percentage of Hexagonal Cells (%HEX)

In the group of patients with type 2 diabetes, a significant correlation was found between cumulative dispersed energy and the percentage of hexagonal cells in the measurement taken 3 months after surgery (*p* = 0.030). The strength of this correlation was low, and the direction was positive (rho = 0.24), meaning that higher values of cumulative dispersed energy were associated with a higher percentage of hexagonal cells, although this relationship was weak. In the remaining measurements in the group of patients with type 2 diabetes, no significant correlation between the analyzed parameters was confirmed (*p* > 0.05).

In the control group, a significant correlation was observed between cumulative dispersed energy and the percentage of hexagonal cells only in the measurement taken 3 months after surgery (*p* = 0.031). The strength of this correlation was low, and the direction was negative (rho = −0.24), meaning that higher values of cumulative dispersed energy were associated with a lower percentage of hexagonal cells, though this relationship was weak. For the remaining measurements in the group of patients without type 2 diabetes, no significant correlation was found between the analyzed parameters (*p* > 0.05 in each case).

**Figure 9:**
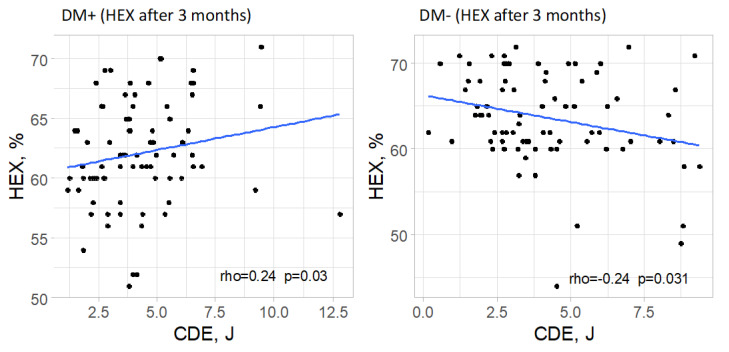
Significant relationships between cumulative dispersed energy (CDE) and the percentage of hexagonal cells (%HEX) in both groups.

Significant correlations are visually presented in Figure 9.

#### 3.7.6. Central Corneal Thickness (CCT)

No significant correlations were observed for any of the measurements in either of the analyzed groups (*p* > 0.05 in all cases).

### 3.8. Analysis of the Correlation Between Cumulative Dispersed Energy (CDE) and Lens Thickness (L) in Cataract

For each group, it was examined whether there was a correlation between cumulative dispersed energy and lens thickness, and if so, what its direction and strength were. Spearman’s correlation analysis was used for this purpose, as both analyzed variables had distributions deviating from normality in the diabetes group. In the control group, these variables had distributions consistent with normality; however, for methodological consistency, Spearman’s correlation analysis was also applied. It was determined that there was no significant correlation between cumulative dispersed energy and lens thickness in either group (*p* = 0.768 and *p* = 0.106 for the diabetes and non-diabetes groups, respectively), as shown in Table 13.

## 4. Results Summary

**Figure 10:**
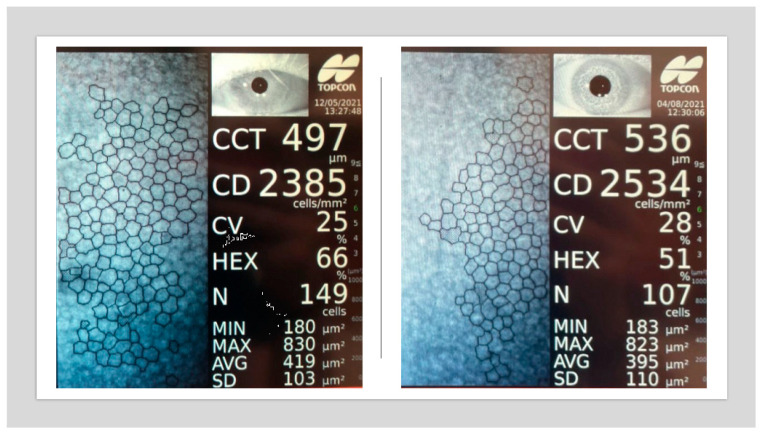
Specular microscopy images showing corneal endothelium of a non-diabetic patient (**left**) and a patient with type 2 diabetes diagnosed 9 years ago (**right**). Note the decreased %HEX and presence of pleomorphism (original data).

**Figure 11:**
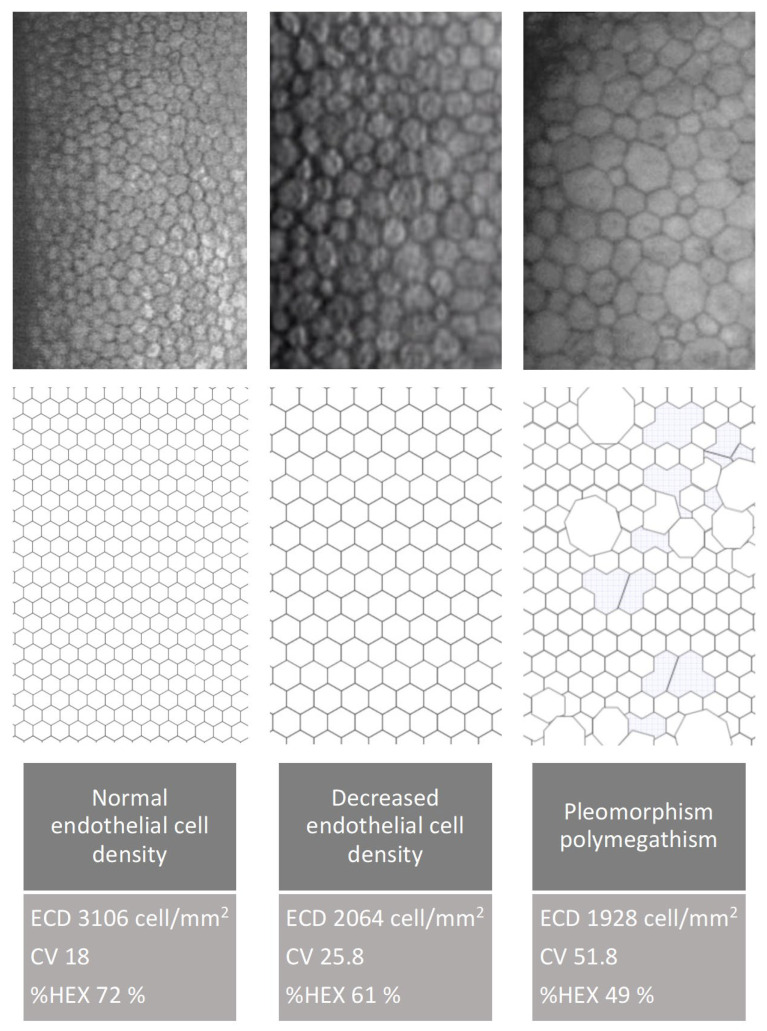
Endothelial cell changes demonstrated using specular microscopy and schematic illustrations. The figure shows a normal endothelial pattern, reduced cell density, pleomorphism, and polymegathism (original data).

Endothelial Cell Density (ECD) in unoperated eyes is lower in the group of patients with well-controlled type 2 diabetes compared to the group of non-diabetic patients. The parameters describing corneal endothelial cell morphology—percentage of hexagonal cells (%HEX) and coefficient of variation (CV)—do not significantly differ between the groups (Figure 10 and Figure 11).

Central corneal thickness (CCT) in unoperated eyes in the group of patients with type 2 diabetes is comparable to the CCT values in unoperated eyes of non-diabetic patients.

After cataract removal surgery using the phacoemulsification method, endothelial cell density (ECD) decreases in both the group of patients with type 2 diabetes and the group of non-diabetic patients. The absolute decline in ECD is similar in both groups. However, when expressed as a percentage of baseline values, the decline is greater in the group of patients with type 2 diabetes compared to the non-diabetic group. In the postoperative period, endothelial cell density remains lower in the group of patients with type 2 diabetes compared to the non-diabetic group.

The percentage of hexagonal cells (%HEX) decreases after cataract removal surgery using phacoemulsification in both diabetic and non-diabetic patients. However, the decline is more pronounced in the group of patients with type 2 diabetes.

The coefficient of variation (CV) of endothelial cells increases in both groups after cataract removal surgery using phacoemulsification. However, this increase is more pronounced in the early postoperative period in the group of patients with type 2 diabetes.

Central corneal thickness (CCT) increases after cataract removal surgery using phacoemulsification in both diabetic and non-diabetic patients. In the early postoperative period, the increase in CCT is more pronounced in the group of diabetic patients. Over a few months of follow-up, CCT returns to baseline values in both groups.

Lower preoperative visual acuity values correspond to higher values of cumulative dispersed ultrasound energy (CDE) in both diabetic and non-diabetic patients.

Higher values of cumulative dispersed ultrasound energy (CDE) correspond to lower endothelial cell density (ECD) values in the postoperative period in both groups—diabetic and non-diabetic patients.

## 5. Discussion

### 5.1. Changes in Corneal Parameters in the Group of Patients with Type 2 Diabetes After Cataract Removal Surgery Using the Phacoemulsification Method

The aim of the present study was to evaluate changes in the corneal endothelium following uncomplicated cataract surgery performed using the phacoemulsification technique. Previous studies addressing this issue have reported inconsistent findings. However, many of these investigations did not account for several preoperative and postoperative factors that may influence outcomes. Key variables often omitted include cataract severity, glycemic control (as measured by HbA1c), duration and type of diabetes (type 1 vs. type 2), presence of diabetic retinopathy, and cumulative dissipated energy (CDE) during surgery. Variability in results may also be attributed to small sample sizes and ethnic differences. Additionally, most prior studies assessed endothelial changes at only a single postoperative time point.

#### 5.1.1. Visual Acuity (Vis)

In this study, preoperative visual acuity was significantly lower in patients with type 2 diabetes compared to the control group. Following cataract surgery via the phacoemulsification technique, both groups demonstrated improvement in visual acuity at each postoperative follow-up. At the 14-day follow-up, visual acuity in the diabetic group remained slightly lower than in the control group; however, this difference was not statistically significant. Notably, this disparity coincided with a more pronounced increase in central corneal thickness (CCT) in the diabetic group, suggesting more severe postoperative corneal edema. Clinically, this may reflect transient endothelial dysfunction, as the corneal endothelium plays a key role in maintaining stromal hydration through its pump function.

Visual acuity continued to improve at later follow-up visits (3 and 6–8 months postoperatively) in both groups. These improvements paralleled changes in CCT: a subclinical increase in CCT was observed at 3 months, followed by a return to baseline values by the 6–8-month mark. The stabilization of visual acuity at these time points corresponded with normalization of CCT, further supporting the association between corneal hydration status and visual recovery.

#### 5.1.2. Endothelial Cell Density (ECD)

In this study, the preoperative endothelial cell density (ECD) was significantly lower in patients with type 2 diabetes than in the control group. At all postoperative follow-up points, both groups showed a significant decline in ECD compared to baseline values. However, comparison of the changes in ECD (ΔECD) between the groups at each follow-up revealed no statistically significant differences in absolute values.

When expressed as a percentage of baseline, the ECD decrease was greater in the diabetic group (18.44%) compared to the control group (15.12%). The lack of statistical significance in absolute ΔECD between the groups may be attributed to the comparable cataract severity (nuclear density < grade 5 on the LOCS III scale) and similar levels of cumulative dissipated energy (CDE) during surgery. Importantly, all diabetic participants had well-controlled glycemia.

Several studies have investigated corneal endothelial cell loss in diabetic patients undergoing cataract surgery via phacoemulsification. Al-Sharkawy et al. reported similar ECD loss (~8%) in both diabetic and non-diabetic groups [14]. Similarly, Lee et al. found comparable ΔECD values among non-diabetic patients and diabetic patients with varying stages of retinopathy 6 months postoperatively [12].

He et al. retrospectively analyzed 133 eyes and reported a greater ECD reduction in diabetic patients (15%) than in non-diabetic patients (11%). This study also accounted for intraoperative factors (CDE, BSS use, surgical duration) but included both type 1 and type 2 diabetic patients without considering disease duration, and had a relatively short follow-up of 1 month [15].

Langwińska-Wośko et al. observed a greater reduction in ECD in diabetic patients (14%) compared to non-diabetic patients (9%), while Tang et al.’s meta-analysis of 13 studies (1923 eyes) found preoperative ECD to be significantly lower in diabetics, with more pronounced postoperative loss at all time points (up to 3 months) [16,17]. Unlike the present study, those studies did not differentiate patients by diabetes duration, type, or glycemic control.

Sahu et al. compared ECD outcomes between patients with well-controlled type 2 diabetes (HbA1c < 7%) and controls, also considering CDE [18]. Although their follow-up was shorter (up to 3 months), they found a significantly greater ECD loss in the diabetic group. Notably, both their study and the present one showed the greatest ECD decline shortly after surgery, followed by stabilization.

The current findings align with the general trend of postoperative ECD reduction and subsequent stabilization. However, in contrast to Sahu et al., the present study did not find a statistically significant difference in ΔECD between the groups.

Other studies, such as that by Morikubo et al., reported significantly greater ECD loss in diabetic patients [6]. However, their study was limited by a short follow-up (1 month) and a lack of data on glycemic control or retinopathy status, potentially explaining discrepancies with our findings.

Hugod et al. also reported a significantly greater ECD decline in the diabetic group at 3 months, while Beato et al., studying patients with well-controlled type 2 diabetes, found no significant difference in ΔECD between groups—findings consistent with those of the present study [5,19].

#### 5.1.3. Coefficient of Variation of Cells (CV)

In the present study, a significant increase in the coefficient of variation of endothelial cell size (CV) was observed in both the diabetic and control groups at 14 days and 3 months postoperatively. At the 6–8-month follow-up, CV values in the diabetic group remained significantly elevated compared to baseline, while in the control group, they had returned to levels comparable with the preoperative measurement.

This postoperative increase in CV reflects enhanced polymegathism, likely representing an endothelial regenerative response to intraoperative stress. The prolonged elevation of CV in diabetic patients may suggest a delayed or impaired regenerative process in this population. Despite these trends, no statistically significant differences were found between the groups in the magnitude of CV change (ΔCV) at any of the follow-up time points.

Sahu et al. reported similar preoperative CV values in both groups and noted a postoperative increase over a 3-month period, consistent with the current study. However, they observed a smaller increase in the diabetic group, which they interpreted as evidence of a blunted regenerative response. This finding was not replicated in the present study or in other literature [18].

Tang et al. also reported increased CV values postoperatively in both groups. Notably, the increase was significantly greater in diabetic patients at the 1-week and 1-month follow-ups but not at 3 months, suggesting a transient disparity in endothelial response [17].

In contrast, Morikubo et al. and Hugod et al. did not observe significant postoperative changes in CV in either group, which differs from the findings of the present study [6,17]. These discrepancies may be related to differences in follow-up duration, patient selection, or study methodology.

#### 5.1.4. Percentage of Hexagonal Cells (%HEX)

Cataract surgery induces trauma to the corneal endothelium, resulting in decreased endothelial cell density (ECD). To maintain coverage of the posterior corneal surface, surviving cells enlarge, leading to increased variation in cell size and loss of their characteristic hexagonal shape. Over time, with regeneration, endothelial cell morphology—including cell shape and size—tends to return toward preoperative values.

In the present study, no significant differences in %HEX were observed between groups in the preoperative period. Postoperatively, a significant reduction in %HEX was noted in both groups, with the decrease being more pronounced in patients with type 2 diabetes. This more substantial reduction may reflect a delayed or impaired regenerative response in the diabetic group following phacoemulsification.

Sahu et al. reported decreases in %HEX in both diabetic and control groups (4.93% and 5.28%, respectively), though these changes did not reach statistical significance 18. Similar trends were observed by Lee et al. and Morikubo et al.; however, both studies were limited by a short postoperative follow-up (1 month) [6,12]. In contrast, Hugod et al. found a significant reduction in %HEX only in diabetic patients, suggesting a potentially greater vulnerability of the diabetic endothelium. The authors proposed that ethnic and metabolic differences may partly explain the variability in endothelial responses across studies [5].

It is important to note that while ECD remains the primary metric for assessing endothelial cell status, it does not fully capture the dynamic changes involved in endothelial regeneration. In contrast, morphological parameters such as CV and %HEX provide more sensitive indicators of the regenerative processes that occur after cataract surgery.

#### 5.1.5. Central Corneal Thickness (CCT)

At 14 days postoperatively, central corneal thickness (CCT) was significantly elevated in both groups compared to baseline. However, the increase (ΔCCT) was significantly more pronounced in the type 2 diabetes group, indicating greater corneal edema in the early postoperative period.

By the 3-month follow-up, CCT values had decreased in both groups compared to the 14-day measurement, though they remained significantly higher than baseline. At the 6–8-month follow-up, CCT values in both groups had returned to baseline levels, and no significant intergroup differences were observed.

These findings suggest that the greater early postoperative corneal edema observed in diabetic patients, reflected in higher CCT values and slightly reduced visual acuity, was transient. In later follow-ups, both CCT and visual acuity (Vis) values stabilized and equalized between groups. This underscores a reversible, stress-induced endothelial dysfunction rather than permanent damage in diabetic patients with well-controlled glycemia.

CCT and Vis are functional indicators of corneal endothelial status. In this study, higher CCT and lower Vis in the diabetic group at 14 days postoperatively corresponded with early endothelial dysfunction. However, the normalization of these parameters by 3 months and beyond, despite ongoing differences in endothelial morphology (CV and %HEX), supports the conclusion that corneal endothelial integrity was preserved.

These findings are consistent with results from Sahu et al., who observed significantly greater CCT increases in diabetic patients at 1 week postoperatively, with gradual reductions over time and minimal intergroup differences by 3 months [18]. Similar patterns were reported by Morikubo et al., Hugod et al., and Tang et al. [5,6,17]. Tang et al. additionally noted a prolonged elevation in CCT in diabetic patients up to 3 months postoperatively, consistent with the trend observed in the present study.

It is important to note that subclinical endothelial cell changes, such as those reflected in CV and %HEX parameters, may persist even when functional metrics like CCT and Vis return to normal. This highlights the regenerative nature of the endothelial response and suggests that, in patients with well-controlled type 2 diabetes and preserved baseline ECD, the risk of long-term corneal decompensation following phacoemulsification is minimal.

#### 5.1.6. Impact of Cumulative Dissipated Energy (CDE)

In the present study, no statistically significant difference in cumulative dissipated energy (CDE) during phacoemulsification was observed between the type 2 diabetes group and the control group. This finding is consistent with data reported by Tang et al., who also found no intergroup differences in CDE values [17]. However, the literature remains limited regarding the influence of CDE on corneal endothelial parameters.

A significant negative correlation was found between CDE and baseline visual acuity (Vis) in both groups. This suggests that poorer preoperative visual acuity, often due to denser or harder cataracts, was associated with the use of higher ultrasound energy during phacoemulsification.

In both diabetic and non-diabetic patients, CDE was negatively correlated with endothelial cell density (ECD) at each postoperative time point. This indicates that higher ultrasound energy exposure is associated with greater endothelial cell loss, supporting the hypothesis that intraoperative energy use directly impacts endothelial integrity.

CDE did not show a significant correlation with lens thickness in either group, suggesting that lens thickness alone is not a sufficient predictor of energy usage during surgery.

For the percentage of hexagonal cells (%HEX), a correlation with CDE was observed at the 3-month follow-up. In the diabetic group, this was a weak positive correlation, whereas in the control group, a weak negative correlation was observed. Although intriguing, these findings have not been reported in other studies and should be interpreted with caution pending further research in larger cohorts.

Comparisons with the study by Sahu et al. reveal partial alignment. While both studies observed a correlation between CDE and ECD in the control group, Sahu et al. did not find a similar correlation in the diabetic group, contrary to the current study’s findings. Additionally, Sahu et al. reported a positive correlation between CDE and CV changes in the control group, a relationship not confirmed here. Both studies identified a correlation between CDE and %HEX in the control group at 3 months, though the correlation was weak [18].

It is important to highlight that certain intraoperative factors, such as total fluid usage, surgical duration, and anterior chamber depth, were not accounted for in this study. These variables may also influence endothelial outcomes and should be considered in future research.

## 6. Conclusions

Phacoemulsification cataract surgery induces temporary but significant alterations in corneal endothelial structure and function in all patients. In individuals with well-controlled type 2 diabetes, these changes, particularly in early postoperative periods, tend to be more pronounced, manifesting as increased corneal thickness, decreased cell density, and more substantial morphological disruption. However, by 6–8 months postoperatively, most parameters, including visual acuity and central corneal thickness, return to levels comparable with non-diabetic controls.

These findings support the relative safety of phacoemulsification in patients with well-managed type 2 diabetes but underscore the importance of close early postoperative monitoring, particularly in individuals with borderline preoperative ECD. Future studies should further explore long-term endothelial morphology and include broader intraoperative variables to refine risk assessment and postoperative care strategies.

## Figures and Tables

**Table 1:** Inclusion and Exclusion Criteria for Patient Recruitment in the Study.

Inclusion Criteria	Exclusion Criteria
- Informed consent from the patient to participate in the clinical study	- Previous ophthalmic surgeries
- Age 60–80 years	- Complications during cataract surgery or in the perioperative period
- Agerelated cataract at stages qualifying for surgical treatment	- Active inflammatory condition of the visual organ
- In the study group, type 2 diabetes treated with oral medications diagnosed between 1 and 10 years prior	- History of uveitis
- Wellcontrolled diabetes (HbA1c ≤ 7%)	- Use of contact lenses
	- Corneal scars, dystrophies
	- History of ocular trauma
	- Traumatic cataract, congenital cataract
	- Documented history of increased intraocular pressure
	- Glaucoma
	- Pseudoexfoliation syndrome
	- Subluxation of the natural lens
	- Preoperative endothelial cell count < 1500cells/mm^2^
	- Difficulty visualizing endothelial cells in the preoperative period
	- Advanced cataract, grade 5 or 6 nuclear density (LOCS III)
	- Axial hyperopia, Axl < 21.0 mm
	- Axial myopia, Axl > 26 mm
	- Autoimmune diseases other than diabetes
	- Hypersensitivity to the active ingredient or excipients in medications administered during the perioperative period

**Table 2:** Characteristics of the Patient Group with Type 2 Diabetes (DM+).

Variable	n (%)/Mean ± SD	Median (Q1; Q3)	Range
N	80 (100.0)	-	-
Gender			
Female	43 (53.8)	-	-
Male	37 (46.2)	-	-
Age (years)	72.04 ± 5.60	73.00 (67.00; 76.25)	60.00 to 80.00
HbA1c before surgery (%)	6.77 ± 0.13	6.80 (6.70; 6.90)	6.50 to 7.00
HbA1c after 3 months (%)	6.77 ± 0.15	6.80 (6.70; 6.90)	6.00 to 7.00
Biometric parameters			
AXL (mm)	23.33 ± 0.91	23.19 (22.67; 23.73)	21.32 to 26.09
L (mm)	4.83 ± 0.52	4.87 (4.62; 5.18)	2.44 to 5.63
Ac (mm)	3.13 ± 0.46	3.16 (2.78; 3.44)	1.98 to 5.33
Corneal endothelium parameters before surgery			
ECD (cells/mm^2^)	2480.76 ± 303.48	2497.50 (2350.00; 2706.00)	1780.00 to 3011.00
CV	30.00 ± 4.81	29.00 (27.00; 32.00)	22.00 to 45.00
%HEX (%)	68.21 ± 4.93	68.00 (65.75; 71.00)	58.00 to 80.00
Other parameters before surgery			
CCT (μm)	542.22 ± 27.94	539.00 (523.00; 559.00)	461.00 to 612.00
Vis	0.44 ± 0.18	0.40 (0.30; 0.60)	0.10 to 1.00
IOP (mmHg)	15.59 ± 2.84	15.00 (14.00; 18.00)	9.00 to 22.00
CDE (J)	4.19 ± 2.05	3.80 (2.71; 5.20)	1.17 to 12.80

Note: SD—standard deviation, Q1—first quartile, Q3—third quartile.

**Table 3:** Characteristics of the Non-Diabetic Patient Group (DM−).

Variable	n (%)/Mean ± SD	Median (Q1; Q3)	Range
N	80 (100.0)	-	-
Gender			
Female	42 (52.5)	-	-
Male	38 (47.5)	-	-
Age (years)	73.22 ± 4.90	74.00 (70.00; 77.00)	60.00 to 80.00
Blood glucose (mg/dL)	92.90 ± 4.08	92.00 (90.00; 95.25)	79.00 to 100.00
Biometric parameters			
AXL (mm)	23.23 ± 0.76	23.14 (22.69; 23.60)	22.01 to 25.42
L (mm)	4.85 ± 0.43	4.86 (4.58; 5.14)	3.66 to 5.68
Ac (mm)	3.09 ± 0.37	3.05 (2.88; 3.31)	1.94 to 3.96
Corneal endothelium parameters before surgery			
ECD (cells/mm^2^)	2629.64 ± 304.73	2635.50 (2414.25; 2781.75)	1977.00 to 3679.00
CV	29.12 ± 4.14	29.00 (26.00; 32.00)	21.00 to 43.00
%HEX (%)	68.86 ± 4.59	69.00 (66.00; 72.00)	54.00 to 79.00
Other parameters before surgery			
CCT (μm)	548.20 ± 24.84	547.00 (532.75; 566.25)	495.00 to 606.00
Vis	0.50 ± 0.19	0.50 (0.40; 0.60)	0.10 to 1.00
IOP (mmHg)	15.38 ± 3.07	15.00 (13.00; 17.00)	9.00 to 25.00
CDE (J)	4.14 ± 2.24	3.54 (2.68; 5.41)	0.17 to 9.37

Note: SD—standard deviation, Q1—first quartile, Q3—third quartile.

**Table 4:** Comparison of Patients with Type 2 Diabetes (DM+) and Without Type 2 Diabetes (DM−) Before Cataract Surgery.

Variable	DM+	DM−	MD (95% CI)	*p*
**Gender**				
Female	43 (53.8)	42 (52.5)	-	>0.999 ^3^
Male	37 (46.2)	38 (47.5)	-	-
**Age (years)**	72.04 ± 5.60	73.22 ± 4.90	−1.19 (−2.83; 0.46)	0.155
**Biometric parameters**				
AXL (mm)	23.19 (22.67; 23.73)	23.14 (22.69; 23.60)	0.04 (−0.16; 0.31) ^1^	0.592 ^2^
L (mm)	4.87 (4.62; 5.18)	4.86 (4.58; 5.14)	0.00 (−0.13; 0.15) ^1^	0.770 ^2^
Ac (mm)	3.16 (2.78; 3.44)	3.05 (2.88; 3.31)	0.11 (−0.09; 0.17) ^1^	0.593 ^2^
**Corneal endothelium parameters before surgery**				
ECD (cells/mm^2^)	2480.76 ± 303.48	2629.64 ± 304.73	−148.88 (−243.84; −53.91)	0.002
CV	29.00 (27.00; 32.00)	29.00 (26.00; 32.00)	0.00 (−1.00; 2.00) ^1^	0.407 ^2^
%HEX (%)	68.21 ± 4.93	68.86 ± 4.59	−0.65 (−2.14; 0.84)	0.390
**Other parameters before surgery**				
CCT (μm)	542.22 ± 27.94	548.20 ± 24.84	−5.98 (−14.23; 2.28)	0.155
Vis	0.44 ± 0.18	0.50 ± 0.19	−0.06 (−0.11; 0.00)	0.049
IOP (mmHg)	15.59 ± 2.84	15.38 ± 3.07	0.21 (−0.71; 1.14)	0.650
CDE (J)	3.80 (2.71; 5.20)	3.54 (2.68; 5.41)	0.26 (−0.49; 0.73) ^1^	0.660 ^2^

Note: Data are presented as the number of observations (% of the group) for categorical variables, mean ± standard deviation for numerical variables with a normal distribution, or median (first quartile; third quartile) for numerical variables with a non-normal distribution. MD—difference in means or medians ^1^ (DM+ vs. DM−), CI—confidence interval. Groups were compared using Student’s *t*-test for independent groups, Mann–Whitney U test ^2^, or Pearson’s chi-square test ^3^.

**Table 5:** Changes in Parameters at Follow-up Visits—Type 2 Diabetes Patient Group.

Variable/Measurement	Mean ± SD	Median (Q1; Q3)	MD (95% CI)	*p*
**Vis**				
Before surgery	0.44 ± 0.18	0.40 (0.30; 0.60)	-	-
After 14 days	0.89 ± 0.15	0.90 (0.80; 1.00)	0.45 (0.40; 0.50)	<0.001
After 3 months	0.97 ± 0.06	1.00 (1.00; 1.00)	0.53 (0.49; 0.57)	<0.001
After 6–8 months	0.97 ± 0.05	1.00 (1.00; 1.00)	0.54 (0.50; 0.58)	<0.001
**ECD (cells/mm^2^)**				
Before surgery	2480.76 ± 303.48	2497.50 (2350.00; 2706.00)	-	-
After 14 days	2027.76 ± 536.38	2170.50 (1781.50; 2415.25)	−327.00 (−477.50; −250.50) ^1^	<0.001 ^2^
After 3 months	2019.75 ± 534.79	2172.50 (1754.75; 2407.75)	−325.00 (−482.50; −263.50) ^1^	<0.001 ^2^
After 6–8 months	2013.00 ± 534.98	2159.50 (1744.50; 2399.00)	−338.00 (−490.50; −269.00) ^1^	<0.001 ^2^
**CV**				
Before surgery	30.00 ± 4.81	29.00 (27.00; 32.00)	-	-
After 14 days	35.08 ± 5.54	35.00 (31.00; 38.00)	6.00 (4.50; 5.50) ^1^	<0.001 ^2^
After 3 months	34.09 ± 5.49	34.50 (31.00; 37.00)	5.50 (4.00; 5.00) ^1^	<0.001 ^2^
After 6–8 months	30.73 ± 4.75	31.00 (28.00; 33.00)	2.00 (0.00; 2.00) ^1^	0.025 ^2^
**%HEX (%)**				
Before surgery	68.21 ± 4.93	68.00 (65.75; 71.00)	-	-
After 14 days	61.19 ± 4.62	61.00 (58.00; 64.00)	−7.00 (−7.00; −6.00) ^1^	<0.001 ^2^
After 3 months	62.05 ± 4.33	62.00 (60.00; 65.00)	−6.00 (−6.50; −5.50) ^1^	<0.001 ^2^
After 6–8 months	65.26 ± 3.93	65.00 (63.00; 68.25)	−3.00 (−3.50; −2.50) ^1^	<0.001 ^2^
**CCT (μm)**				
Before surgery	542.22 ± 27.94	539.00 (523.00; 559.00)	-	-
After 14 days	563.02 ± 28.84	561.50 (543.50; 583.75)	22.50 (18.00; 22.00) ^1^	<0.001 ^2^
After 3 months	546.56 ± 26.21	541.50 (529.00; 564.25)	4.34 (2.67; 6.01)	<0.001
After 6–8 months	542.79 ± 27.44	540.00 (524.00; 557.50)	0.56 (−0.66; 1.78)	0.361

Note: Data are presented as mean ± standard deviation and median (first quartile; third quartile). MD—difference in means or medians ^1^ (subsequent measurement vs. preoperative measurement), CI—confidence interval. Each postoperative measurement was compared to the preoperative measurement using paired *t*-tests or Wilcoxon tests ^2^.

**Table 6:** Changes in Parameters at Follow-up Visits—Non-Diabetic Patient Group (DM−).

Variable/Measurement	Mean ± SD	Median (Q1; Q3)	MD (95% CI)	*p*
**Vis**				
Before surgery	0.50 ± 0.19	0.50 (0.40; 0.60)	-	-
After 14 days	0.92 ± 0.12	1.00 (0.90; 1.00)	0.43 (0.38; 0.47)	<0.001
After 3 months	0.96 ± 0.06	1.00 (0.90; 1.00)	0.47 (0.43; 0.51)	<0.001
After 6–8 months	0.97 ± 0.05	1.00 (0.98; 1.00)	0.48 (0.44; 0.52)	<0.001
**ECD (cells/mm^2^)**				
Before surgery	2629.64 ± 304.73	2635.50 (2414.25; 2781.75)	-	-
After 14 days	2236.94 ± 505.27	2275.00 (2031.00; 2576.25)	−360.50 (−450.50; −258.50) ^1^	<0.001 ^2^
After 3 months	2203.60 ± 513.56	2241.50 (1988.00; 2535.00)	−394.00 (−485.50; −285.00) ^1^	<0.001 ^2^
After 6–8 months	2195.09 ± 512.31	2231.00 (2015.75; 2496.00)	−404.50 (−498.50; −295.00) ^1^	<0.001 ^2^
**CV**				
Before surgery	29.12 ± 4.14	29.00 (26.00; 32.00)	-	-
After 14 days	33.66 ± 5.18	33.00 (30.00; 36.00)	4.00 (3.50; 5.00) ^1^	<0.001 ^2^
After 3 months	33.05 ± 4.68	33.00 (29.00; 36.00)	4.00 (3.50; 4.50) ^1^	<0.001 ^2^
After 6–8 months	29.82 ± 6.03	29.00 (27.00; 32.00)	0.00 (−0.50; 1.50) ^1^	0.242 ^2^
**%HEX (%)**				
Before surgery	68.86 ± 4.59	69.00 (66.00; 72.00)	-	-
After 14 days	63.09 ± 5.71	63.00 (59.00; 67.00)	−6.00 (−6.50; −4.50) ^1^	<0.001 ^2^
After 3 months	63.71 ± 5.34	64.00 (61.00; 68.00)	−5.00 (−6.00; −4.00) ^1^	<0.001 ^2^
After 6–8 months	66.55 ± 5.67	67.00 (64.00; 70.00)	−2.00 (−3.00; −1.00) ^1^	<0.001 ^2^
**CCT (μm)**				
Before surgery	548.20 ± 24.84	547.00 (532.75; 566.25)	-	-
After 14 days	562.71 ± 30.40	562.50 (542.00; 579.50)	15.50 (10.00; 15.50) ^1^	<0.001 ^2^
After 3 months	549.16 ± 23.17	551.00 (533.00; 566.25)	4.00 (0.50; 3.00) ^1^	0.009 ^2^
After 6–8 months	547.19 ± 22.49	548.00 (531.00; 564.50)	1.00 (−1.50; 1.00) ^1^	0.714 ^2^

Note: Data are presented as mean ± standard deviation and median (first quartile; third quartile). MD—difference in means or medians ^1^ (subsequent measurement vs. preoperative measurement), CI—confidence interval. Each postoperative measurement was compared to the preoperative measurement using paired *t*-tests or Wilcoxon tests ^2^.

**Table 7:** Comparison of patients with diabetes (DM+) and without diabetes (DM−) in terms of studied parameters at different follow-up points.

Variable/Measurement	DM+	DM−	MD (95% CI)	*p*
**IOP, mmHg**				
Before surgery	15.59 ± 2.84	15.38 ± 3.07	0.21 (−0.71; 1.14)	0.650
After 14 days	14.14 ± 2.16	13.89 ± 2.37	0.25 (−0.46; 0.96)	0.486
After 3 months	13.00 (12.00; 15.00)	14.00 (12.00; 15.00)	−1.00 (−1.00; 1.00) ^1^	0.967 ^2^
After 6–8 months	13.26 ± 1.70	13.39 ± 1.85	−0.12 (−0.68; 0.43)	0.657
**Vis**				
Before surgery	0.44 ± 0.18	0.50 ± 0.19	−0.06 (−0.11; 0.00)	0.049
After 14 days	0.90 (0.80; 1.00)	1.00 (0.90; 1.00)	−0.10 (−0.22; −0.04) ^1^	0.086 ^2^
After 3 months	1.00 (1.00; 1.00)	1.00 (0.90; 1.00)	0.00 (0.00; 0.00) ^1^	0.464 ^2^
After 6–8 months	1.00 (1.00; 1.00)	1.00 (0.98; 1.00)	0.00 (0.00; 0.00) ^1^	0.682 ^2^
**ECD, cells/mm^2^**				
Before surgery	2480.76 ± 303.48	2629.64 ± 304.73	−148.88 (−243.84; −53.91)	0.002
After 14 days	2027.76 ± 536.38	2236.94 ± 505.27	−209.17 (−371.90; −46.45)	0.012
After 3 months	2019.75 ± 534.79	2203.60 ± 513.56	−183.85 (−347.58; −20.12)	0.028
After 6–8 months	2013.00 ± 534.98	2195.09 ± 512.31	−182.09 (−345.65; −18.52)	0.029
**CV**				
Before surgery	29.00 (27.00; 32.00)	29.00 (26.00; 32.00)	0.00 (−1.00; 2.00) ^1^	0.407 ^2^
After 14 days	35.00 (31.00; 38.00)	33.00 (30.00; 36.00)	2.00 (0.00; 3.00) ^1^	0.035 ^2^
After 3 months	34.50 (31.00; 37.00)	33.00 (29.00; 36.00)	1.50 (0.00; 3.00) ^1^	0.086 ^2^
After 6–8 months	31.00 (28.00; 33.00)	29.00 (27.00; 32.00)	2.00 (0.00; 3.00) ^1^	0.055 ^2^
**%HEX, %**				
Before surgery	68.21 ± 4.93	68.86 ± 4.59	−0.65 (−2.14; 0.84)	0.390
After 14 days	61.00 (58.00; 64.00)	63.00 (59.00; 67.00)	−2.00 (−4.00; 0.00) ^1^	0.016 ^2^
After 3 months	62.00 (60.00; 65.00)	64.00 (61.00; 68.00)	−2.00 (−3.00; 0.00) ^1^	0.011 ^2^
After 6–8 months	65.00 (63.00; 68.25)	67.00 (64.00; 70.00)	−2.00 (−3.00; 0.00) ^1^	0.010 ^2^
**CCT, μm**				
Before surgery	542.22 ± 27.94	548.20 ± 24.84	−5.98 (−14.23; 2.28)	0.155
After 14 days	561.50 (543.50; 583.75)	562.50 (542.00; 579.50)	−1.00 (−8.00; 10.00) ^1^	0.735 ^2^
After 3 months	546.56 ± 26.21	549.16 ± 23.17	−2.60 (−10.32; 5.12)	0.507
After 6–8 months	540.00 (524.00; 557.50)	548.00 (531.00; 564.50)	−8.00 (−13.00; 2.00) ^1^	0.185 ^2^

Note: Data are presented as mean ± standard deviation for numerical variables with a normal distribution or as median (first quartile; third quartile) for variables with a non-normal distribution. MD—difference in means or medians ^1^ (DM+ vs. DM−), CI—confidence interval. Groups were compared using the Student’s *t*-test for independent groups or the Mann–Whitney U test ^2^.

**Table 8:** Changes in Analyzed Parameters Between Follow-Up and Baseline Measurements for Patients with Type 2 Diabetes.

Variable/Measurement	Mean ± SD	Median (Q1; Q3)	Range
**Vis**			
After 14 days	−0.45 ± 0.21	−0.40 (−0.60; −0.30)	−0.90 to 0.10
After 3 months	−0.53 ± 0.18	−0.50 (−0.70; −0.40)	−0.90 to 0.00
After 6–8 months	−0.54 ± 0.18	−0.50 (−0.70; −0.40)	−0.90 to 0.00
**ECD, cells/mm^2^**			
After 14 days	453.00 ± 468.37	233.50 (156.50; 572.75)	45.00 to 1960.00
After 3 months	461.01 ± 466.09	239.00 (177.50; 552.50)	50.00 to 1979.00
After 6–8 months	467.76 ± 466.27	252.50 (148.50; 607.50)	48.00 to 1981.00
**CV**			
After 14 days	−5.08 ± 4.23	−4.00 (−7.00; −3.00)	−18.00 to 15.00
After 3 months	−4.09 ± 4.06	−4.00 (−6.00; −3.00)	−13.00 to 16.00
After 6–8 months	−0.72 ± 3.87	−1.00 (−3.00; 1.00)	−12.00 to 14.00
**%HEX, pp**			
After 14 days	7.03 ± 3.85	6.00 (5.00; 8.00)	1.00 to 19.00
After 3 months	6.16 ± 3.11	6.00 (4.00; 7.25)	−1.00 to 17.00
After 6–8 months	2.95 ± 3.79	3.00 (1.00; 4.00)	−8.00 to 14.00
**CCT, μm**			
After 14 days	−20.80 ± 9.87	−20.50 (−25.00; −13.00)	−59.00 to −2.00
After 3 months	−4.34 ± 7.50	−4.00 (−9.00; 2.00)	−23.00 to 10.00
After 6–8 months	−0.56 ± 5.47	−1.00 (−4.00; 2.00)	−18.00 to 17.00

Note: SD—standard deviation, Q1—first quartile, Q3—third quartile.

**Table 9:** Changes in Analyzed Parameters Between Follow-Up and Baseline Measurements for Patients Without Type 2 Diabetes.

Variable/Measurement	Mean ± SD	Median (Q1; Q3)	Range
**Vis**			
After 14 days	−0.43 ± 0.20	−0.40 (−0.50; −0.30)	−0.90 to 0.10
After 3 months	−0.47 ± 0.19	−0.50 (−0.60; −0.40)	−0.90 to 0.00
After 6–8 months	−0.48 ± 0.19	−0.50 (−0.60; −0.40)	−0.90 to 0.00
**ECD, cells/mm^2^**			
After 14 days	392.70 ± 411.23	228.50 (73.75; 615.00)	−115.00 to 1643.00
After 3 months	426.04 ± 418.95	276.50 (83.00; 661.25)	−90.00 to 1670.00
After 6–8 months	434.55 ± 419.70	272.50 (94.25; 650.00)	−59.00 to 1664.00
**CV**			
After 14 days	−4.54 ± 3.77	−4.00 (−6.00; −3.00)	−19.00 to 4.00
After 3 months	−3.92 ± 3.37	−4.00 (−6.00; −3.00)	−15.00 to 5.00
After 6–8 months	−0.70 ± 4.77	−1.00 (−2.00; 2.25)	−23.00 to 12.00
**%HEX, pp**			
After 14 days	5.78 ± 5.32	5.00 (3.00; 7.00)	−9.00 to 27.00
After 3 months	5.15 ± 5.23	4.00 (3.00; 7.00)	−6.00 to 30.00
After 6–8 months	2.31 ± 5.66	2.00 (−1.00; 4.25)	−8.00 to 31.00
**CCT, μm**			
After 14 days	−14.51 ± 18.08	−11.50 (−20.00; −6.00)	−100.00 to 38.00
After 3 months	−0.96 ± 8.98	−2.00 (−5.00; 1.25)	−20.00 to 55.00
After 6–8 months	1.01 ± 8.25	0.00 (−2.00; 3.00)	−19.00 to 53.00

Note: SD—standard deviation, Q1—first quartile, Q3—third quartile.

**Table 10:** Comparison of patients with diabetes (DM+) and without diabetes (DM−) in terms of the difference in the magnitude of change in analyzed parameters relative to the baseline measurement.

Variable/Measurement	DM+	DM−	MD (95% CI)	*p*
**Vis**				
After 14 days	−0.45 ± 0.21	−0.43 ± 0.20	−0.02 (−0.09; 0.04)	0.489
After 3 months	−0.53 ± 0.18	−0.47 ± 0.19	−0.06 (−0.12; −0.01)	0.029
After 6–8 months	−0.54 ± 0.18	−0.48 ± 0.19	−0.06 (−0.11; 0.00)	0.048
**ECD, cells/mm^2^**				
After 14 days	233.50 (156.50; 572.75)	228.50 (73.75; 615.00)	5.00 (−25.00; 128.00) ^1^	0.161 ^2^
After 3 months	239.00 (177.50; 552.50)	276.50 (83.00; 661.25)	−37.50 (−50.00; 113.00) ^1^	0.285 ^2^
After 6–8 months	252.50 (148.50; 607.50)	272.50 (94.25; 650.00)	−20.00 (−40.00; 107.00) ^1^	0.242 ^2^
**CV**				
After 14 days	−4.00 (−7.00; −3.00)	−4.00 (−6.00; −3.00)	0.00 (−1.00; 0.00) ^1^	0.133 ^2^
After 3 months	−4.00 (−6.00; −3.00)	−4.00 (−6.00; −3.00)	0.00 (−1.00; 0.00) ^1^	0.327 ^2^
After 6–8 months	−1.00 (−3.00; 1.00)	−1.00 (−2.00; 2.25)	0.00 (−1.00; 1.00) ^1^	0.513 ^2^
**%HEX, pp**				
After 14 days	6.00 (5.00; 8.00)	5.00 (3.00; 7.00)	1.00 (0.00; 2.00) ^1^	0.021 ^2^
After 3 months	6.00 (4.00; 7.25)	4.00 (3.00; 7.00)	2.00 (0.00; 2.00) ^1^	0.012 ^2^
After 6–8 months	3.00 (1.00; 4.00)	2.00 (−1.00; 4.25)	1.00 (0.00; 2.00) ^1^	0.106 ^2^
**CCT, μm**				
After 14 days	−20.50 (−25.00; −13.00)	−11.50 (−20.00; −6.00)	−9.00 (−11.00; −4.00) ^1^	<0.001 ^2^
After 3 months	−4.00 (−9.00; 2.00)	−2.00 (−5.00; 1.25)	−2.00 (−4.00; 0.00) ^1^	0.055 ^2^
After 6–8 months	−1.00 (−4.00; 2.00)	0.00 (−2.00; 3.00)	−1.00 (−2.00; 0.00) ^1^	0.177 ^2^

Note: Data are presented as mean ± standard deviation or median (first quartile; third quartile), depending on normality of distributions. MD—difference in means or medians ^1^ (DM+ vs. DM−), CI—confidence interval. Comparisons were made using Student’s *t*-test for independent groups or the Mann–Whitney U test ^2^.

**Table 11:** Characteristics of Endothelial Cell Density (ECD) Change in Both Groups.

ECD Change, %/Measurement	Mean ± SD	Median (Q1; Q3)	Range
**DM+**			
After 14 days	−18.44 ± 18.49	−9.65 (−22.18; −6.04)	−68.63 to −1.84
After 3 months	−18.77 ± 18.37	−9.86 (−21.82; −6.88)	−69.29 to −1.83
After 6 months	−19.05 ± 18.39	−10.20 (−23.49; −6.17)	−69.36 to −1.97
**DM−**			
After 14 days	−15.12 ± 16.13	−9.07 (−22.73; −2.92)	−63.27 to 4.35
After 3 months	−16.42 ± 16.61	−10.31 (−24.12; −3.45)	−64.30 to 3.41
After 6 months	−16.73 ± 16.61	−10.74 (−23.62; −3.85)	−64.07 to 2.23

Note: The ECD change is expressed as a percentage relative to the baseline measurement for each follow-up point. SD—standard deviation, Q1—first quartile, Q3—third quartile.

**Table 12:** Results of the correlation analysis between cumulative dispersed energy (CDE) and parameters for both analyzed groups.

Measurement	Vis		ECD, Cells/mm^2^		CV		%HEX, %		CCT, µm	
	rho	*p*	rho	*p*	rho	*p*	rho	*p*	rho	*p*
**DM+**										
Before surgery	−0.36	0.001	-	-	-	-	-	-	-	-
After 14 days	0.07	0.522	−0.41	<0.001	−0.09	0.405	0.12	0.292	−0.01	0.963
After 3 months	0.11	0.323	−0.41	<0.001	−0.22	0.051	0.24	0.030	−0.04	0.732
After 6 months	0.08	0.458	−0.42	<0.001	−0.13	0.239	0.20	0.076	−0.05	0.629
**DM−**										
Before surgery	−0.35	0.002	-	-	-	-	-	-	-	-
After 14 days	−0.09	0.403	−0.38	0.001	−0.04	0.745	−0.19	0.094	−0.12	0.285
After 3 months	0.12	0.304	−0.37	0.001	−0.02	0.829	−0.24	0.031	−0.14	0.207
After 6 months	0.03	0.817	−0.37	0.001	−0.03	0.774	−0.14	0.201	−0.13	0.238

Note: rho—Spearman’s correlation coefficient.

**Table 13:** Results of the correlation analysis between cumulative dispersed ultrasound energy (CDE) and lens thickness (L) for both analyzed groups.

Group	rho	*p*
**DM+**	0.03	0.768
**DM−**	−0.18	0.106

Note: rho—Spearman’s correlation coefficient.

## Data Availability

The data that support the findings of this study are available from the corresponding author upon reasonable request.
